# Mapping Intestinal Paracellular Perm Eability in Mice: Regional and Cellular Variability Under Physiological and Stimulated Conditions

**DOI:** 10.1096/fba.2025-00325

**Published:** 2026-03-05

**Authors:** Mathilde Miquel, Kadirey Verwaerde, Anissa Edir‐Kibri, Mikael Albin, Florence Blas‐Y‐Estrada, Audrey Samper, Elodie Rousseau‐Bacquie, Hervé Robert, Hélène Eutamène, Vassilia Théodorou, Christine Coméra

**Affiliations:** ^1^ Toxalim, Toulouse University, INRAE, ENVT, EI‐Purpan Toulouse France; ^2^ INSERM, UMR‐1297 and Université Toulouse III, Institut de Maladies Métaboliques et Cardiovasculaires (I2MC), CHU‐Rangueil Toulouse France; ^3^ Toulouse University, IRSD, INSERM, INRAE, ENVT Toulouse France

**Keywords:** chronic stress, enteroendocrine cells, goblet cells, intestine, lipid absorption, paracellular permeability, tuft cells

## Abstract

Intestinal paracellular permeability was analyzed ex vivo by incubation of tissue segments at 0°C with the fluorescent dyes FM1‐43FX (FM) or TRITC‐dextran 3 kDa lysine‐fixable (TD3L) and confocal microscopy in (i) healthy mice and (ii) mice submitted to chronic stress or lipid diets. In the small intestine of healthy mice, FM staining was restricted to the apical surface of enterocytes but fully penetrated around Goblet cells, enteroendocrine cells, tuft cells, and apoptotic cells. The same cell types were similarly labeled in the colon when located on the tissue surface but not within the crypts. TD3L exhibited a comparable labeling pattern but also showed moderate staining of the basolateral surface of enterocytes at the tips of small intestinal villi, and also substantial penetration around colonic epithelial cells at the surface or top of crypts. The study reveals patterns of permeability likely corresponding to the “leak” pathway of paracellular transport through the intestinal epithelium, because transcellular endocytosis is blocked at 0°C. This pathway is found around specific cell populations involved in the luminal detection of food, antigens, microbes, or their secretions. These trigger immune, neural, and tissue responses that maintain intestinal homeostasis. Chronic stress induced by glucocorticoid exposure increased FITC‐dextran 4 kDa permeability in vivo. Using FM, increased paracellular permeability was also detected ex vivo and selectively localized in the colon of stressed mice. A single oral administration of a lipid‐rich food also increased ex vivo permeability around jejunal enterocytes. Pathophysiological increases in paracellular permeability are therefore detectable using the FM methodology.

## Graphical abstract

This study analyzed the paracellular permeability of the intestines of healthy mice at a basal level. A new detection method involving tissue incubation for 5 min with luminal fluorescent dyes, such as FM1‐43FX (FM), which bind to accessible plasma (or external) cell membranes, was developed. Using confocal microscopy, we identified certain epithelial cells, such as goblet cells, as being naturally permeable to FM, while others were impermeable. This selective passage may belong to the leak intestinal paracellular pathway.

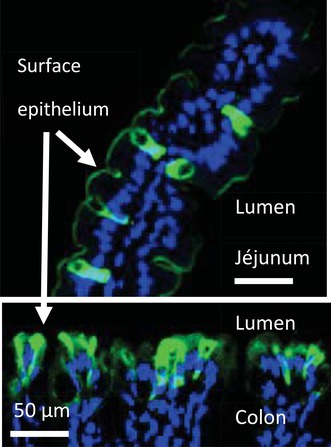

**Abbreviations**
CCh: carbamylcholine chloride
DAPI: 4',6‐diamidine‐2'‐phenylindole dihydrochloride
DCAMKL 1: doublecortin and calcium/calmodulin‐dependent protein kinase‐like‐1
FD4: FITC‐labeled dextran 4 kDa
FITC: fluorescein isotiocyanate
FM: FM1‐43FX
GAP: goblet associated passage
GC: goblet cells
IEC: intestinal epithelial cells
LPS: lipopolysaccharide
PBS: phosphate buffered saline
TD3L: TRITC‐dextran 3 kDa lysine‐fixable
TiO_2_
: titanium dioxide
TJ: tight junction
TRITC: *Tetramethylrhodamine‐isothiocyanate*
WGA: wheat germ agglutinin
ZO: zonula occludens

## 1 Introduction

The intestinal barrier consists of a monolayer of intestinal epithelial cells (IECs) which form the first line of defense between the internal environment and the gut lumen. Along with the outer mucus layer and the internal immune, blood and nerve protection systems of the gut, the epithelium ensures the absorption of nutrients, drugs and other xenobiotics, while maintaining a constant protective barrier against harmful luminal agents. Exchanges across the monolayer are highly regulated and involve either paracellular transport through the intercellular space or transcellular transport within the IEC by the absorption and, often, transcytosis of various components, such as nutrients, antigens and xenobiotics, as well as microorganisms [1, 2, 3].

Intestinal epithelial cells (IECs) comprise various cell types, including stem cells, Paneth cells, microfold cells (M cells), enterocytes, enteroendocrine cells, tuft cells, and Goblet cells or GC. Apical (tight junctions (TJ), adherens junctions, and desmosomes) connect all of these cells via their lateral membranes. These form a cohesive structure, the opening of which is regulated by TJs. The diameter of the paracellular pore pathway, which transports ions and solutes, ranges up to 8 Å, while the leak pathway, which ensures conjoint non‐specific uptake of larger molecules including nutrients and antigens, ranges up to 125 Å [1, 4, 5]. TJs are composed of various proteins; the three main transmembrane proteins are occludin, claudins, and junction adhesion molecule proteins. These are associated with cytosolic proteins, such as zonula occludens (ZO), which are attached to the actin cytoskeleton.

The regulation of paracellular transport is crucial to the maintenance of intestinal homeostasis. Larger opening of the TJ or epithelial disruption leads to increased intestinal permeability which is often associated to digestive and extra‐digestive diseases [1, 4, 5, 6, 7, 8].

The transcellular transport pathway allows various apical or basolateral absorptions in IEC of various components or microbes. It is regulated or not by specific plasma membrane receptors leading to active uptake, endocytosis or transcytosis of luminal xenobiotics, nutrients by enterocytes [9] or antigens and microbiota mainly carried out either by M cells in lymphoid organs such as Peyer's patches or by GC in intestinal villi or colonic crypts, in a process known as Goblet cell associated passage (GAP) [3, 10, 11]. In reference to immunoglobulin A notably, transcellular transport also occurs from the basolateral pole of the epithelium to the intestinal lumen [12].

Different methods have been developed to measure paracellular intestinal permeability from decades either in vivo, ex vivo or in vitro using biopsies, tissue fragments or monocellular cultured epithelium or organoids. For in vivo assays, small molecules are administered orally in the form of non‐metabolized carbohydrates (e.g., mannitol or lactulose), fluorescent probes (e.g., FITC or fluorescein isotiocyanate‐dextran 4 kDa) or radioactive probes (e.g., Cr^51^‐EDTA) [7, 8, 13, 14, 15]. Paracellular permeability is measured by determining the concentration of these substances in the blood or urine after they cross the intestinal epithelium. ELISA identification of several biomarkers which are normally present in the intestinal lumen as LPS (lipopolysaccharide) or in the epithelium as LPS‐binding protein, intestinal fatty‐acid binding protein, zonulin in the blood stream or urine is also used to determine paracellular permeability disturbances [7, 16]. However, despite the different sizes of these global probes and biomarkers, there is no convincing evidence of their passage into the intestine, either through paracellular pathways (pore or leak) or after disruption of TJ, or even by transcellular transport [7, 17].

Using the Ussing's chamber technique overcomes some of these limitations. The technique allows paracellular flux to be measured across TJ. This makes it possible to more accurately evaluate either pore‐ or leak‐mediated paracellular pathways, or non‐specific and unrestricted diffusion, across isolated gut segments [1, 18]. However, this technique only provides an overall view of paracellular permeability levels but does not permit the characterization of the paracellular permeability flux according to the type of the intestinal epithelial cells considered. Beside, confocal or intravital microscopy approaches using fluorescent membrane markers (including the FM‐1‐43‐FX (FM)), antigens or microorganisms have been used to characterize the endocytic transcellular Goblet associated passage [3, 19, 20, 21]. These studies are performed with cells or tissues incubated at 37°C where endocytic and exocytotic processes occur. The characterization of the intestinal paracellular pathway using confocal microscopy and fluorescent probes across cultivated epithelial monolayers, organoids, or biopsies has begun, but remains unclear, especially throughout the entire intestine [21, 22, 23, 24, 25]. Based on these observations, the aim of this study was to determine intestinal paracellular permeability patterns using confocal microscopy in basal conditions and in mice subjected to chronic stress or a lipid‐enriched diet. Knowing that endocytosis is blocked at temperatures below 10°C [20, 26, 27], we introduced fluorescent dyes in the lumen of intestinal fragments and incubated them at 0°C. The aim was to detect any potential paracellular uptake between epithelial cells by confocal microscopy.

## 2 Materials and Methods

### 2.1 Ethics Approval for Animal Models

All animal experiments adhered to the guidelines outlined in European legislation (Council Directive 2010/63/UE) and French Decree 2013‐118 concerning the welfare of animals used for scientific purposes. Approval for the experiments was obtained from the Local Animal Care and Ethics Committee (CEEA‐86 Toxcomethique, authorization number 2021040710365316) under agreement CEEA. They were performed on male C57BL/6 mice (Janvier Laboratories, RRID:IMSR_JAX:000664) aged 8 to 15 weeks, assigned to 12/12 h of light/dark from 8 h A.M. at a 22°C + 2 housing temperature and were raised with constant access to food (Teklad Global 18% protein rodent diet) and water, except in cases where fasting was necessary prior to experimentation. The animals were treated humanely, with due consideration given to the need to minimize distress, discomfort, and injury. The number of animals used was reduced to a minimum of 8 animals, assigned in random allocation, while ensuring that the batches were sufficient to obtain valid statistical analyses.

### 2.2 Ex Vivo Labeling of Intestinal Isolated Fragments

The mice were used for experiments in basal and stimulated conditions as previously described and were euthanized by cervical dislocation [25]. For all the ex vivo experiments, fresh jejunal or colonic segments were gently managed, washed with 0.9% NaCl, cooled to 0°C on ice, ligatured at one extremity and filled with 1 μg/mL solution of FM‐1‐43FX. FM is a non‐permeable dye, which selectively binds into phospholipids of the outer leaflet of plasma membranes accessible from the gut lumen. The second tissue end was ligated and incubated for 5 min at 0°C in 0.9% NaCl. The ligatures were then cut to remove the luminal dye. The tissue was then filled with cold 8% paraformaldehyde and incubated in this fixative for 16 h.

Under basal conditions, tissue segments were treated and harvested as described above, followed by a 15‐min incubation at 0°C with 0.5 mg/mL of TRITC (*Tetramethylrhodamine‐isothiocyanate*)‐labeled 3‐kDa lysin‐fixable dextran (T3DL) in PBS and then fixed with paraformaldehyde. T3DL is water‐soluble and can enter extracellular spaces. The fixative then attaches T3DL to the surface of adjacent cells via its free lysine end.

Fixed tissues were incubated for 24 h in 30% sucrose in phosphate buffered saline (PBS), were embedded in Neg‐50, frozen in liquid nitrogen, and stored at −80°C. Next, tissue samples were cryo‐sectioned at 14 μm thickness.

Tissue sections were rehydrated and permeabilized in PBS containing 0.2% Triton X100 for 10 min, then incubated for 30 min with 1 μg/mL AF‐647 WGA in the same buffer. After three washings of 5 min, the sections were finally mounted with fluorescent mounting medium with DAPI (4‐6 diamino‐2‐phenylindole, Origen E1918).

The labeling of the fluorescent markers was imaged using a laser‐scanning confocal microscope Leica TCS SP8 (Leica, Nanterre, France).

### 2.3 Cholinergic Regulation of GC Paracellular Permeability

Previous studies described that cholinergic receptors activate transcellular passage through GC named GAP but also mucus secretion [19]. We therefore checked whether, under our conditions, the regulation of paracellular passage was also controlled by muscarinic receptors. Three groups of mice were used: (i) 6 mice treated with 125 μg/kg of the receptor agonist carbamylcholine chloride or CCh (Sigma‐Aldrich, France) administered subcutaneously 1 h before intestinal segments harvest, (ii) 6 mice receiving 550 μg/kg of the antagonist atropine (Sigma‐Aldrich, St Louis, MO) administered intraperitoneally during 30 min before intestinal segments harvest and (iii) 6 control mice receiving vehicle. The fresh intestinal segments were removed to be labeled ex vivo with FM‐1‐43FX.

### 2.4 Chronic Stress Experiments

To analyze the effect of chronic stress on intestinal permeability, 3.5 mg of corticosterone was solubilized in 10 mL of 44% β‐cyclodextrin by sonication and completed to 1 L with drinking water for final concentrations of 3.5 μg/mL cortisone and 4.4 g/L cyclodextrin. A fresh solution is produced every 3 days. It was given to drink to 8 mice of the CORT group for 24 days [28, 29] while 8 control (CTRL) mice were given tap water. On day 21, the in vivo intestinal permeability was analyzed in mice that had been starved for 14 h, before receiving an oral administration of 150 μL of 150 mg/mL FITC‐dextran 4 kDa (FD4) [14, 30]. Two hours later, 10 μL of blood was collected from the tail and added to a saline solution containing 1000 IU/mL of heparin. This measures the paracellular passage of the dye through the intestinal epithelium into the blood. Fluorescence intensity was measured using a Smart microplate spectrophotometer (Tecan) at excitation 488 nm/emission 525 nm and expressed as ng of FD4/mL of blood.

On day 24, the mice were weighed and sacrificed, and the jejunal and colonic segments were isolated and labeled with FM, as described below. Using confocal microscopy, the number of cells labeled by FM at the surface of the epithelial monolayer, as well as the number of cells with a deeper location in the tissue, was determined by double‐blind counting in each tissue. The deeper FM penetration was representative of paracellular permeability and was scored as a percentage of the total number of apical and deep cells.

### 2.5 Lipid Diets Experiments

The effect of dietary lipid absorption was studied using three types of single feed administered to mice after 14 h of fasting, divided into different groups of eight mice. The first meal was an oral gavage at *t* = 0 h of a 200 μL emulsion. This emulsion was half aqueous, containing 35% (w/w) sucrose, 21% (w/v) protein and 4% (w/w) cellulose fibers, as found in generic mouse kibble. The other half was lipidic, containing either olive or palm oil, corresponding to 46% (w/w). All of these were final concentrations. The controls received the same nutrients but without lipids. Thirty min after feeding (*t* = 0.5 h), mice received an oral administration of FD4 (150 μL at 150 mg/mL) and the in vivo paracellular permeability to this marker was evaluated 2 h later (*t* = 2.5 h) as described above. Finally, at *t* = 3.5 h after feeding, the mice were sacrificed at *t* = 3.5 h where lipid absorption is maximal [31] and their proximal jejunum was harvested for ex vivo FM labeling to evaluate intestinal permeability.

The second diet consisted of feeding 8 mice conventional mouse kibble (Inotiv Diet 2018, France) containing 6% (W/W) lipids. For the third diet for 8 mice, the same kibble was ground, enriched with olive oil to 46% (W/W) total lipids, then reconstituted. Both kibble types were given at *t* = 0 h then removed at *t* = 2 h and weighed to measure food consumption. Height control mice remained starved. The mice were then sacrificed at *t* = 3.5 h after the start of feeding and their proximal jejunal segments were harvested for ex vivo evaluation of FM labeling. In vivo permeability was not investigated after kibble feeding to avoid absorption interference between FD4 and the food bolus in the stomach or proximal intestine.

### 2.6 Immunostaining and Lipid Droplet Labeling

For immunostaining, intestine cryosections from 4 mice were permeabilized at 20°C for 10 min in PBS (phosphate buffered saline), 0.2% Triton X100, washed 3 times in PBST (PBS, 0.02% Tween‐20) and saturated in PBST plus 0.5% Triton X100 and 1% bovine serum albumin for 1 h. They were then incubated overnight at 4°C in PBST, 0.5% Triton X100 with the following rabbit polyclonal antibodies directed to either: chromogranin A (CGA) diluted at 1/400 (Abcam ab15160, RRID:AB_301704), mucin 2 (muc 2) at 1/100 (Santacruz Biotechnology sc15334, USA, RRID:AB_2146667), DCAMKL1 at 1/200 (calcium/calmodulin‐dependent protein kinase‐like‐1, Abcam ab‐31704, RRID:AB_873537) or caspase 3 at 1/200 (Cell Signaling Technology 9664, RRID:AB_2070042). Tissues were then washed 3 times in PBST and incubated for 45 min in anti‐rabbit IgG linked to the fluorophore DyLight 594 diluted at 1/200 (Diagomics: DkxRb‐003‐E594NHSX). After 3 washes in PBST the tissue slides were mounted in the fluorescent mounting medium. Anti‐muc 2 immunostaining was completed by AF 647 WGA incubation at 1 µg/ml to compare the two labels.

In several cryosections, lipid droplets were stained with 1 ng/mL LD540 at which stain lipid droplets (a gift from Christophe Thiele, Bonn, Germany). After three washings of 5 min, the sections were finally mounted with fluorescent mounting medium with DAPI (4‐6 diamino‐2‐phenylindole, Origen E1918).

Tissues were imaged using confocal microscopy to detect the cells labeled with FM‐1‐43‐FX, WGA, LD540, or specific antibodies. Concomitantly, tissue structure was detected by brightfield observation using differential interference contrast (DIC). Using three different sequence analyses to avoid fluorescence overlap, DAPI was visualized with laser excitation at 405 nm and detection at 420–480 nm, FM was excited at 488 nm and visualized at 500–600 nm, DyLight 594 and TD3L were excited at 552 nm and visualized between 560 and 610 nm, and WGA 647 was excited at 638 nm and visualized between 650 and 690 nm.

### 2.7 Statistical Analysis

A number of 8 mice were used for each statistical analysis. To analyze the statistical differences between two groups, a Mann–Whitney test was used, and differences among three groups were analyzed using one‐way ANOVA (GraphPad Prism, version 6.0; GraphPad Software, La Jolla, CA, USA, RRID:SCR_002798). *p*‐values of 0.05 or less were considered statistically significant.

## 3 Results

### 3.1 Imaging of Intestinal Paracellular Permeability Patterns in Basal Conditions

**FIGURE 1:**
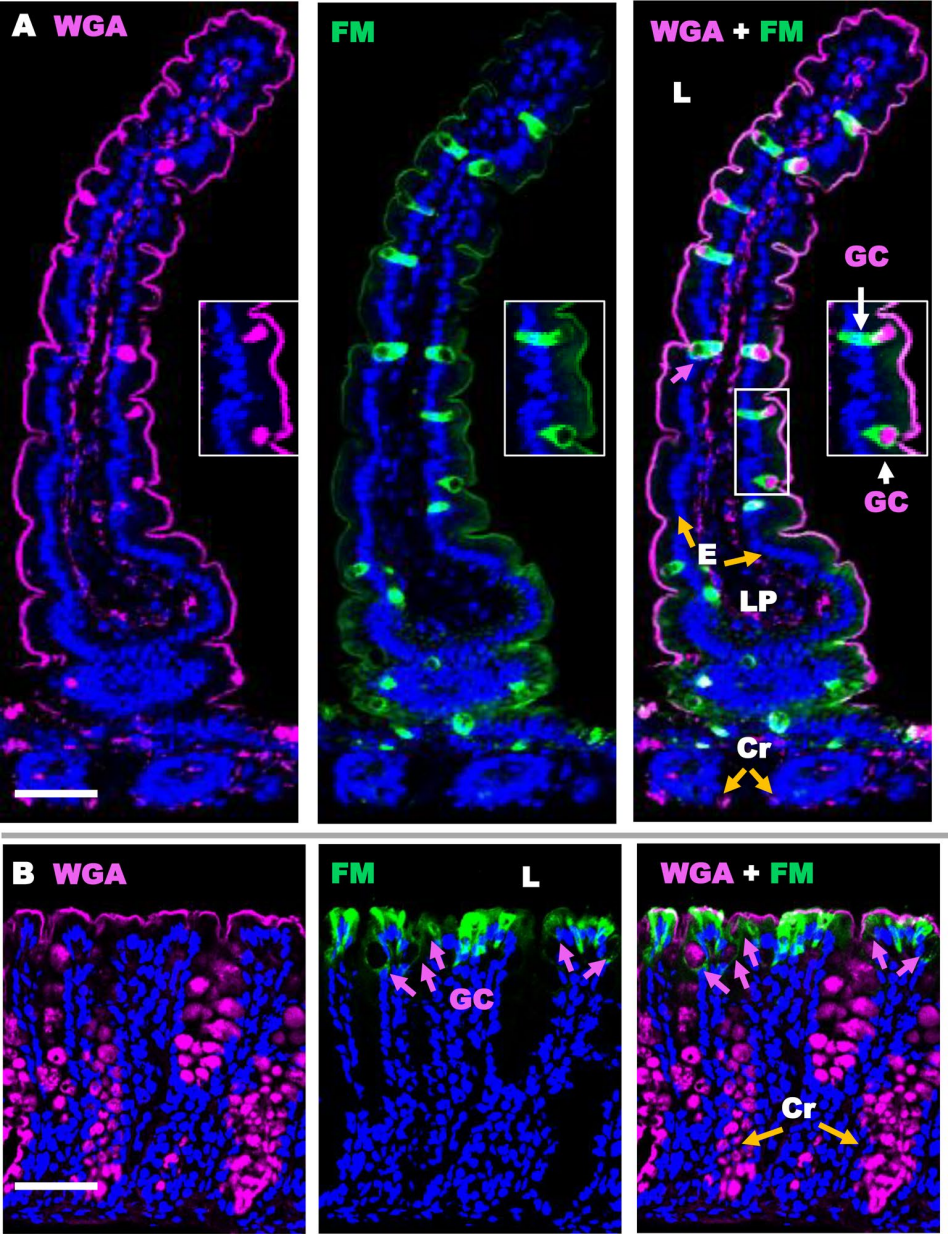
Localization of FM distribution in jejunal villi (A) and colon (B) using confocal microscopy. FM dye appears green, WGA appears cyan in the glycoproteins of plasma membranes and mucus on the surface or within GC cells, and the nucleus is stained blue with DAPI. FM is visible on the apical surface of the intestinal epithelium, but has also penetrated around certain epithelial cells, including GC. The GC appears either full (white arrows) or depleted of mucus (cyan arrows). Cr = crypt as indicated with orange arrows, E = enterocytes, L = lumen, LP = lamina propria. Bars 50 μm.

**FIGURE 2:**
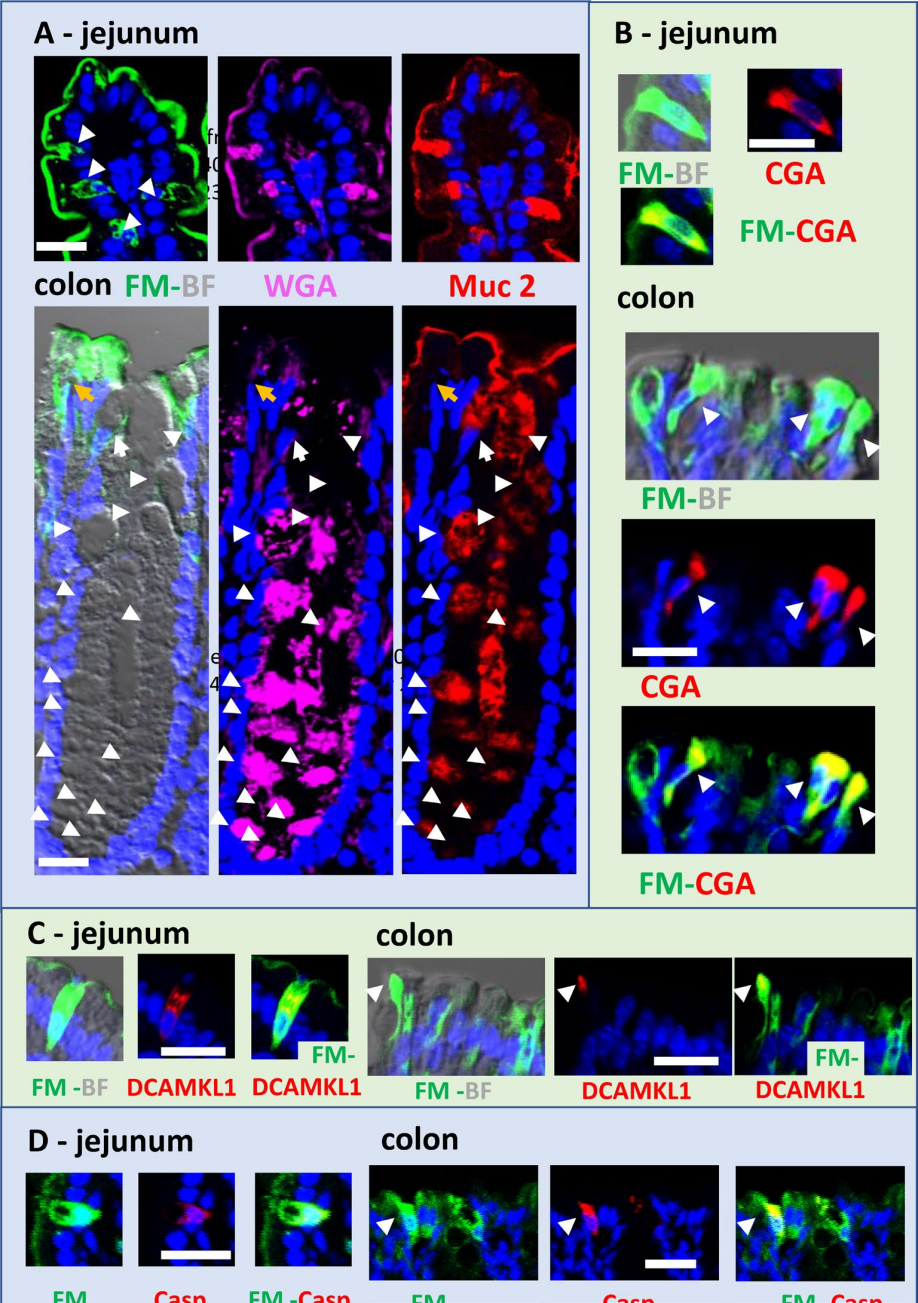
Immunodetection was used to identify permeable epithelial cells in the jejunum or colon that were stained by the FM dye (green). This revealed cell expressing GC mucin 2 (A), chromogranin A‐containing enteroendocrine cells (B), DCAMLCK1‐containing tuft cells (C) and caspase 3‐expressing apoptotic cells (D), which were stained red. The mucin 2 and the WGA colocalized in the tissue surface and into CG (A). Bars = 20 μm. FM (green), WGA (cyan), immunodetection (red) and nucleus (DAPI blue). Bars = 20 μm.

Paracellular permeability was studied using low temperature of incubation, 0°C, because endocytosis is totally inhibited at temperatures lower than 10°C results in a decrease in phospholipid fluidity, which rigidifies the membrane and blocks endocytosis [26, 27]. Membranes accessible to the gut lumen were labeled with FM because the dye is not cell permeable, lipophilic, and binds to the outer leaflet of accessible cell plasma membranes. For most of the epithelium, especially in the small intestine villi, the FM dye only stained the apical but not the basolateral membranes (Figure 1). This is particularly clear for enterocytes (shown by white arrows) and reflects a blockage of FM entry through the apico‐lateral TJ all along jejunal villi. On the contrary, and rather surprisingly, several cells are totally marked by FM, both apically and basolaterally. These include GC, distinguished by their globular structures containing secretory vesicles, the majority of which are filled with mucus and stained with WGA and co‐labeled with anti‐muc 2 antibodies (Figure 2A). The lectin stains mucus and other glycoproteins in apical epithelial membranes, as well as plasma membranes inside the intestine. For example, it highlights the basolateral membranes of the epithelium and cellular surface in the lamina propria. Note that the FM dye does not penetrate the lumen of small intestinal crypts, showing no labeling even on the surface of the local epithelium. In the colon, several cells of the surface epithelium are fully marked by FM, including spherical GC that are more or less filled with mucus, as well as other elongated epithelial cells. In contrast, cells located deeper in the colonic crypts were not labeled, including many WGA positive GC. Finally, the FM binding of lymphoid organs of the entire intestine as Peyer's patches remains strikingly on the apical surface, indicating no paracellular permeability (not shown). To validate our method physiologically, we verified that similar FM labeling patterns were observed when intestinal fragments were incubated at 20°C or 37°C (not shown). These similar staining profiles can be explained by the fact that a 5 min incubation is too short to observe endocytosis at 37°C, which requires an incubation of 30 to 60 min [19, 20, 26, 27]. In any case, it is certain that incubations at 0°C do not involve endocytosis.

In addition to GC, other cells paracellularly labeled with FM were identified using immunodetection with specific epithelial markers. These include enteroendocrine cells that express chromogranin A (Figure 1B), and tuft cells that contain DCAMKL1, whose characteristic apical tuft is clearly visible (Figure 1C). A few scattered apoptotic epithelial cells, mainly GC, were labeled with anti‐caspase 3 antibodies in the middle or apex of the jejunal villi, and on the apical surface of the colon (Figure 1D).

**FIGURE 3:**
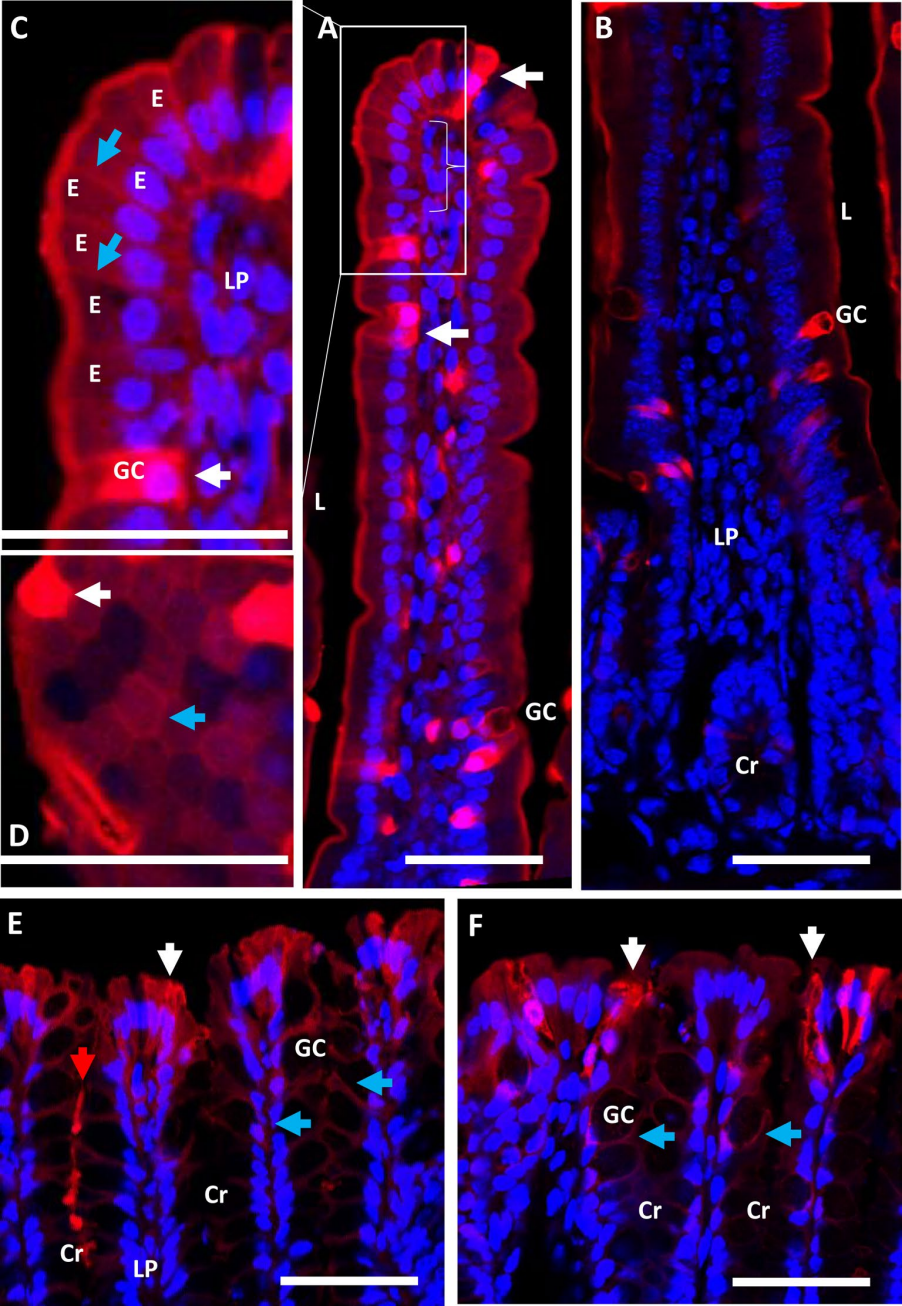
Identification of the TDL3 uptake in the jejunum (A–D) and colon (E, F). The TDL3 shows similar extensive labeling of several epithelial cells (see white arrows) being GC in majority as visible in red while the nucleus in blue. All images showed lateral sections of the intestine except (D) showing a cross section of a jejunal villus. TDL3 is also detected in lower intensity (see blue arrows) around enterocytes in the top of jejunal villi and around several colonocytes. The red arrow shows luminal entry of TDL3 in a colon crypt. Cr = crypt, E = enterocyte, GC = goblet cell, L = lumen, LP = lamina propria. Bars 50 μm.

We also conducted ex vivo permeability experiments using TRITC dextran 3 kDa lysine (TD3L) dye. The paracellular input of the TD3L is similar to that of the FD4, which is used in conventional in vivo measurements. However, its lysine residue enables fixation to adjacent proteins during fixation with paraformaldehyde, allowing tissue localization via fluorescence microscopy. As shown in Figure 3, TD3L strongly penetrates around permeable cells, similar to FM labeling. In the small intestine, however, additional, weaker staining was detected penetrating along the basolateral membrane of enterocytes at the top of several villi (Figure 3A,C), while fading gently downward (Figure 3B). A cross‐section of the intestinal monolayer of the upper villus (Figure 3D) shows similar TD3L staining, very high for the most permeable cells, weaker around the lateral membranes of most enterocytes, but also absent for a few others. In the colon epithelium (Figure 3E,F), strongly positive cells are surrounded by other moderately stained cells, either on the surface of the tissue or deeper in the crypts. TD3F also penetrates into the lumen of one crypt (Figure 3E).

Both dyes are similarly detectable inside the highly positive cells, even though the matrices have not been absorbed. This may be due to dye smearing during fixation or confocal examination.

### 3.2 Regulation of GC Paracellular Permeability by Cholinergic Receptors

The regulation of FM paracellular permeability by cholinergic receptors has been studied according to [19], given that they activate both the degranulation of GC and the opening of GAPs. Quantification of GC/jejunal villus number and their potential for FM staining shows that the majority of GCs are labeled by FM in tissues from control mice, as well as from mice treated with either agonist CCh or antagonist atropine (see Figure S1), underlining the absence of cholinergic regulation of paracellular FM permeability.

### 3.3 Intestinal Paracellular Permeability Patterns in Chronic Stress Conditions

**FIGURE 4:**
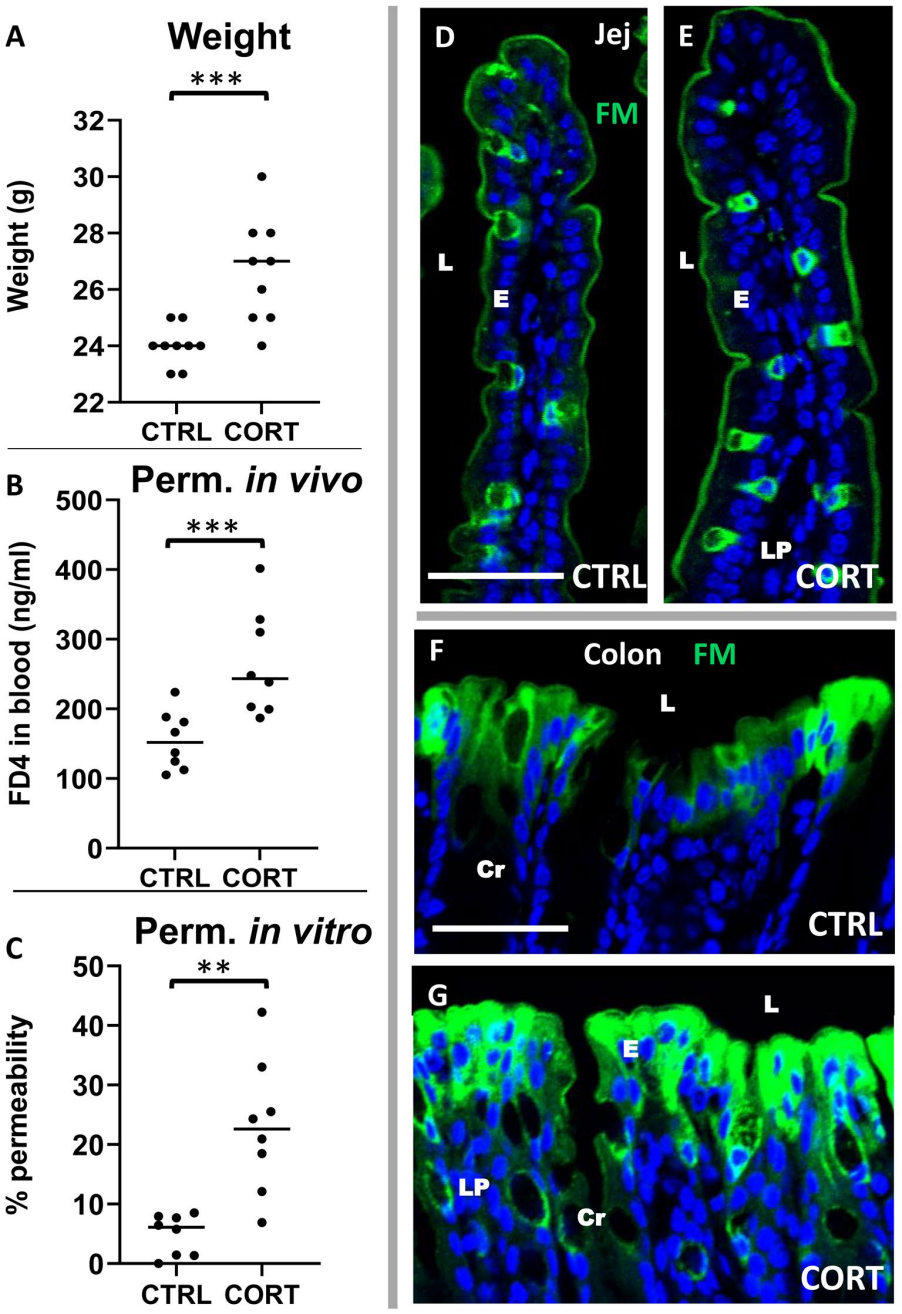
Effect of chronic psychological stress in corticoid treated mice (CORT) versus controls (CTRL). Compared to controls, CORT mice showed significative differences either in weight gain (A), increased in permeability in vivo (B) or ex vivo in the colon (C). The confocal imaging of FM uptake in the jejunum (Jej, D for CTRL, E for CORT) does not shown increased permeability whereas such increase is clearly visible in the CORT (G) versus CTRL (F) colon. FM (green) and DAPI (blue). Cr = crypt, E = enterocytes, L = lumen, LP = lamina propria. Bars 50 μm. Significative differences: ***p < 0.002; **p < 0.02.

In the study of psychological stress induced by glucocorticoids, the CORT group showed a significant increase in weight on day 21 (mean 26.9 g ± 0.5 SEM) compared to the water‐drinking control mice (CTRL, mean 24.0 g ± 0.2, *p* = 0.0018, *p* < 0.002, Figure 4A) as previously reported [29]. In vivo permeability was evaluated at day 22 on starved mice, force fed with FD4. Two hours later, a blood sample was taken. FD4 concentration was analyzed by fluo‐spectrophotometry showing a significant increase in FD4 concentration in the blood of CORT‐treated mice (mean 264 ± 26.9 ng/mL) compared to CTRL mice (mean 155 ± 14.8 ng/mL, *p* = 0.0002; see Figure 4B).

Ex vivo permeability experiments were performed on day 24 in the jejunum and colon of each mouse. No increase in permeability was observed in the jejunum of CORT mice compared to CTRL (Figure 4D). By contrast, paracellular uptake of FM increased in the colon of CORT mice, spreading from the surface epithelium to the adjacent intra‐tissue cells. In CTRL, it was restricted to surface cells. In CTRL (Figure 4E,F). We counted the FM‐labeled cells present on the surface epithelium and the inner ones, located beneath it. To determine the degree of paracellular permeability, we calculated the percentage of internal cells as a proportion of all labeled cells (apical and deeper). A significant increase in permeability was observed in CORT mice (mean 22.9% ± 3.1) compared to CTRL mice (mean 4.9% ± 1.2, *p* = 0.021) (see Figure 4C). Our results indicate that chronic stress increases paracellular permeability, as detected in vivo and ex vivo. This increase is localized to the colon using the latest FM detection technique.

### 3.4 Intestinal Paracellular Permeability Patterns During Lipid Absorption

**FIGURE 5:**
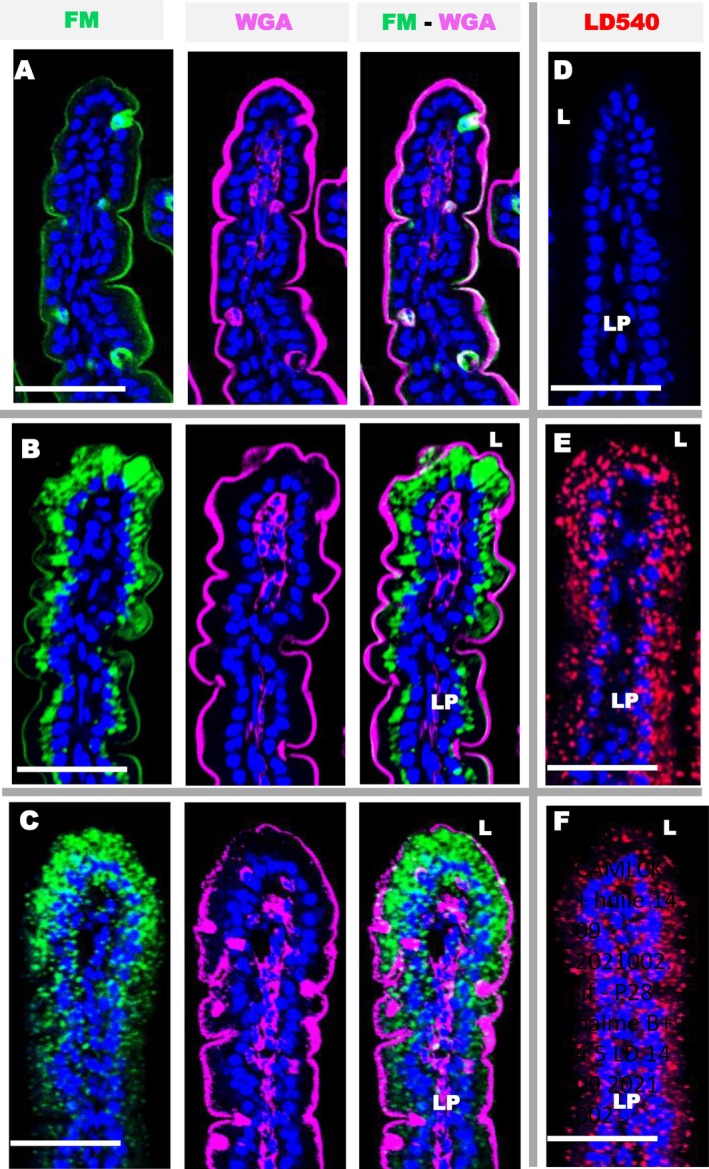
Detection of the uptake FM and lipids in the jejunum after gavage with control solution lipid free (A, D), or emulsions containing olive (B, E) or palm oil (D, F). (A–C) Staining with FM (green), WGA (magenta) and DAPI (blue) show an important uptake of the FM around enterocytes showing increased permeability after olive or palm oil nutrition. In control mice, the villus is devoid of lipid droplets (D), while gavage with olive (E) or palm oil (F) show the presence of numerous lipid droplets stained with LD540 (red), mainly in enterocytes. DAPI stained nucleus in blue. L = lumen, LP = lamina propria. Bars 50 μm.

In mice receiving a single oral administration of a liquid mixture of sugar, proteins and cellulose supplemented with 46% P/V of olive or palm oil we observed numerous lipid droplets stained with the LD 540 in fixed tissue slices (Figure 5E,F). An important penetration of FM1 43‐FX added for 5 min in the lumen of living jejunum, before fixation was also observed across the epithelium, and especially in the top of villi (Figure 5B,C). These two distinct stains are located in the same areas of the intestinal epithelium and show significant lipid uptake and a concomitant increase in intestinal paracellular permeability. As expected, the intestine of control mice showed no lipid droplets or FM permeability, except around highly permeable cells (Figure 5A,B). The permeability to FD4 was also evaluated in vivo after gavages. Compared to control mice with an average of 219.14 ng of FD4 per mL of blood (±27.65 SEM), we observed a non‐significant increase in intestinal paracellular permeability after the administration of olive oil (435.3 ng/mL ± 123.87) and palm oil (433.26 ng/mL ± 72.90, *p* = 0.013). Eight hours after oil administration, the jejunum no longer contains any lipid droplets and the impermeability of the enterocytes was completely restored (not shown).

**FIGURE 6:**
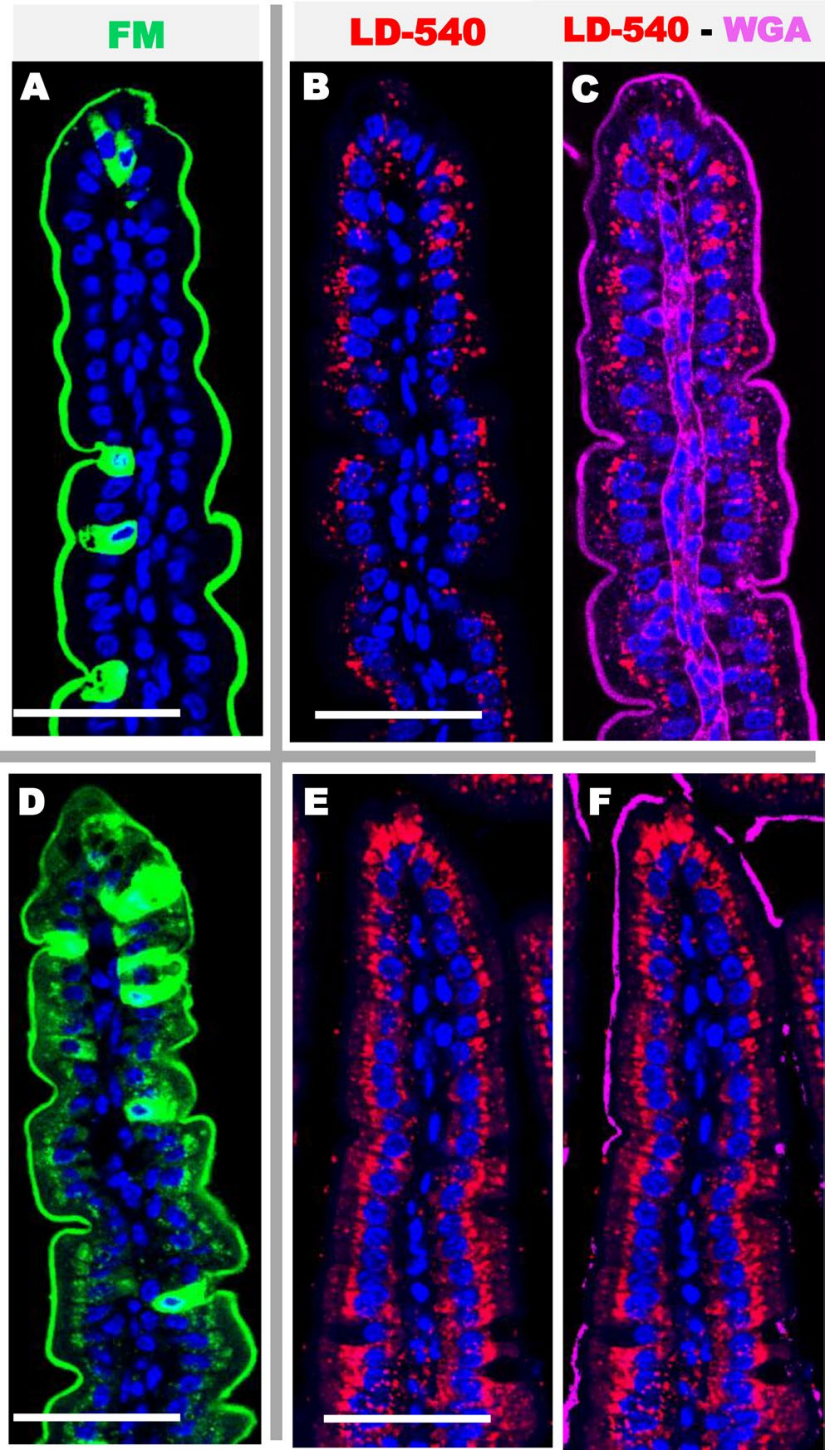
Confocal detection of the uptake of FM (green) or lipids in the jejunum 3.5 h after ingestion of conventional kibble (6% fat W/W) (A–C) or kibble enriched to 46% fat (D–F). Low fat diet provides no increase in permeability (B) compared to controls (A) and moderate content of lipid droplets (E) labeled with LD540 in red. Fat enriched kibble diet induces moderate increase in permeability across enterocytes (C) and shows the presence of lipid droplets (F). WGA (magenta) DAPI (blue). L = lumen, LP = lamina propria. Bars 50 μm.

Therefore, we wanted to find out whether an increase in paracellular permeability could be caused by a normal diet consisting of basic kibble or kibble enriched with olive oil. We found that the content of lipid droplets was highest 3.5 h after feeding began. During the 2 h of feeding, the mice ingested between 0.50 and 1.2 g of nutrients (average of 0.73 g ± 0.27), which represented 15% of their daily intake. At 3.5 h after a conventional diet (6% fat), the jejunum showed no increase permeability to FM outside the GC (Figure 6A), and the amount of fat ingested was close to 7% of the fat administered by gavage. Animals fed pellets enriched with 46% fat ingested approximately 40% of the total lipids administered by liquid gavage. Depending on the mouse, their jejunal villi showed no increase in permeability or moderate permeability to FM between enterocytes (Figure 6B), in addition to permeable goblet‐like cells. The presence of lipid droplets was the greatest 3.5 h after feeding in both cases then gradually decreased (see Figure 6B,C,E,F).

## 4 Discussion

The aim of our study was to characterize intestinal paracellular permeability patterns in mice across the entire length of the gut and according to the different types of epithelial cells. We employed confocal imaging in both basal (healthy) conditions and following chronic stress or a lipid‐rich diet. The FM dye binds on the plasma membrane and is internalized in endocytic vesicles, notably in the intestinal epithelium when incubated at 37°C [20, 25, 32]. But at temperatures below 10°C, when any endocytic process is inhibited, FM binding is restricted to extracellular membranes that are physically accessible to the luminal environment. In this study, enterocytes show a strict impermeability to FM paracellular penetration at low incubation temperature according to [20, 25, 32]. However, we observed that FM penetrated the epithelium of the small intestine and the apical surface of the colon, staining the apical and basolateral membranes of GC, as well as less prevalent cells such as enteroendocrine cells, tuft cells, and apoptotic epithelial cells. The major cells are GC, whose proportion in the epithelium increases from 4% in the jejunal epithelium to 16% in the colon [10, 19, 33], while enteroendocrine cells and tuft cells represent 1% and 0.3%, respectively, distributed throughout the intestine [34, 35]. No permeability was observed by contrast in small intestinal or colonic crypts, possibly due to constraints imposed by the TJ themselves, or due to FM inaccessibility caused by mucus or other secretions obstructing the luminal environment. Likewise, no permeability was observed in the surface of Peyer's patches or colonic lymphoid organs.

Labeling of intestinal segments with TD3L reveals a similar pattern to FM, specifically surrounding certain epithelial cells as GC in the majority of intestinal villi and on the colonic surface. Weaker staining by TD3L but not FM is also visible around enterocytes located at the top of small intestinal villi, showing slight paracellular permeability of differentiated epithelium. TD3L also reveals minor epithelial cell permeability in the colon, either on the surface or deeper in the crypts. This additional uptake can be explained by the hydrophilicity of TD3L, which may allow for a more efficient paracellular propagation than FM binding to membrane lipids. For reasons that we don't understand, these cells are often completely stained, even though we know that the FM and TD3L dyes can't penetrate them under our experimental conditions. The short incubation times of fresh tissue at low temperatures enabled us to rule out the possibility of artificial cytolysis of FM‐permeable cells.

This suggest that, despite the presence of their apico‐lateral proteins, the TJ in GC, enteroendocrine cells, and tuft cells are more permeable structures. Madara and Trier's team used cryogenic scanning electron microscopy to characterize epithelial TJ in rat ileal villi. They described the TJ membrane proteins between enterocytes as being closely juxtaposed and having a uniform structure. These proteins form several strands, each approximately 200 nm thick, that are connected to each other [36]. In comparison, the TJ surrounding goblet and tuft cells are similar in terms of depth and number of horizontal strands, but some appear fragmented. This includes insufficient cross‐linking of strands, abluminal strands with free ends and highly fragmented strands [36, 37]. Some GC also exhibit increased uptake of lanthanum or barium ions, collectively suggesting increased paracellular permeability. These different tight junction configurations can be influenced by either physiological regulation or physical constraints between cells with distinct morphologies and cytoskeletal organizations (e.g., compared to enterocytes). Regarding enteroendocrine cells, it has been demonstrated that they selectively express claudin‐4 in their TJ and are permeable to FITC‐10‐kDa dextran via paracellular or transcellular permeability which is consistent with our results [38]. These observations could ultimately explain the increase in paracellular permeability observed in our study in almost all Goblet, enteroendocrine and tuft cells exposed to the lumen of the entire intestine. Interestingly, these cell types have in common to act as sensors for several luminal signals, including nutrients (such as sugar, amino acid or lipids), the intestinal microbiome or parasites (through interaction or product secretion), antigens or bacterial transcytosis. They then stimulate adaptive or innate immune response and to interact with nerve fibers [3, 39, 40]. Our results suggest that their increased paracellular permeability may contributes to these functions.

Previous studies have described a transcellular absorption of antigens, bacteria, or viruses through GAP's transcytosis associated or not to apical receptor recognition [3, 19, 21, 41, 42, 43, 44] leading to their basolateral delivery to antigen presenting cells (APCs). It represents the main pathway leading to the induction of immunologic tolerance. Cholinergic stimulation of GC was shown to upregulate the transcellular GAP pathway as well as mucus secretion in the small intestine or distal colon. In this study, we demonstrate the absence of cholinergic regulation of paracellular FM absorption. This suggests that, under healthy conditions, both the transcellular and paracellular pathways exist through these cells in both the small intestine and the distal colon. In the latter, distinct localization involving paracellular absorption in surface GCs and GAPs into several crypts can be observed [19].

Based on the results of our ex vivo study, we can conclude that different permeability patterns occur in the gut, with transcellular absorption via GAP and paracellular uptake in several specific epithelial cells. For example, increased paracellular permeability may allow *Listeria monocytogenes* to colonize GC by recognizing its specific receptor, E‐cadherin, which is expressed on the lateral sides of these cells. The bacterium is then rapidly internalized, leading to epithelial transcytosis [45]. We have also shown that dietary TiO_2_ is mainly absorbed in the jejunum via the paracellular route and is often observed in close proximity to GC, which must facilitate its passive lateral entry [25].

The paracellular pathway described in this study would certainly participate to a passive absorption of luminal compounds that could also contribute to intestinal homeostasis and communication to immune cells as well as initial tissue penetration. It has been shown that paracellular absorption involved both a pore and a weak pathway. The pore pathway is a high‐conductance route that is charge and size‐selective, with an upper limit of 6‐ to 8‐Å diameter leading fluid and ion exchange across the epithelium. In contrast, the leak paracellular pathway is less well‐defined either for its localization and is of lower conductance, charge nonselective and with an upper size limit ∼100‐Å diameter. Indeed, it can transport larger molecules as FD4, sugar, peptides both in vivo or in vitro in tissue fragments or cultured epithelial monolayers [1, 4, 5, 8, 14, 15, 16, 17, 18]. Our results suggest that, in the native intestine, most of the leak pathway involves FM‐ and TDL3‐positive paracellular permeability around GC, enteroendocrine and tuft cells. It may also involve lower T3DL uptake around enterocytes in the jejunum and covering several colon IECs. This experiment enables the identification and localization of the paracellular leak pathway, at least in part, as it relates to specific IECs. Figure 3D shows a cross‐sectional view of the epithelial monolayer in the small intestine. TD3L stains the basolateral surface of some enterocytes, reflecting their weak permeability but few enterocytes appear impermeable and unlabeled, indicating pore impermeability. Similar fluorescent labeling of organoids or differentiated epithelial monolayers would permit to precise leak paracellular permeability as previously investigated [22, 24].

Several physiological or pathophysiological conditions such as chronic stress or lipid absorption have been associated with increased intestinal paracellular permeability [30, 46, 47, 48]. We studied these two conditions to determine if our method using FM is able to identify and localize such paracellular permeability.

The induction of chronic stress mimicking psychological stress, induced by corticosterone intestinal delivery in rodents was used herein in order to determine intestinal paracellular patterns in vivo and ex vivo. Three weeks of glucocorticoid treatment leads to significant weight gain [46, 48] and in vivo intestinal paracellular hyperpermeability to FD4 in line with previous studies [46, 48]. Consistent with these works, ex vivo measurements of intestinal permeability to FD4 also show increased paracellular permeability in the colon of stressed mice, but not in the jejunum. Our data emphasize the importance of using fluorescence microscopy to assess paracellular permeability in pathological conditions. This enables the localization of the permeability in the intestine and the identification of the epithelial cells involved.

High fat diet in humans or animals was described as increasing intestinal permeability either directly or because of dysbiosis induction [30, 47]. Previous studies have also reported that the oral administration of 200 μL of palm oil to mice one to five times increased intestinal paracellular permeability to FD4 in vivo, which was associated with decreased tight junction expression [30]. The authors obtained similar results in vitro using Caco‐2 cells that were cultured and supplemented with palmitic acid. These studies prompted us to evaluate the effect on paracellular permeability of the oral administration of a reduced volume of 100 μL of either palm oil or olive oil. This did not result in a significant increase in FD4 permeability in vivo. By contrast, using both oils, ex vivo FM labeling revealed extensive increases in paracellular permeability around the enterocytes and in the lamina propria, particularly in the upper villi. The villi also contained numerous lipid droplets stained with LD540.

Concerning kibble nutrition experiments, mice fed with conventional kibble show no increased permeability except around GC, when compared to controls, and modest content of lipid droplets in enterocytes. A moderate increase in permeability to FM occurs with a fat kibble diet, particularly between enterocytes, but less than following oil force‐feeding. This could be explained by the fact that the mice received about 40% less lipid content with fat kibble than with force‐feeding. Additionally, the liquid nutrients from force‐feeding digest and reach the small intestine more quickly, so they don't get mixed into the food bolus, unlike kibble nutrition. This shows that high delivery of lipid to the jejunum may increase paracellular permeability punctually during food absorption, but moderate delivery does not. It should be noted that paracellular absorption of dietary sugar or amino acids has also been described in the intestinal epithelium, in addition to trans‐enterocyte absorption, and is attributed to a selective response to high luminal intake [18, 49, 50].

In conclusion, this study shows the existence of a natural paracellular permeability of several epithelial cells in the whole healthy intestine which involved in majority a characteristic set of cells being GC, enteroendocrine cells, tuft cells, and apoptotic cells, being permeable to FM and TD3L. Knowing the importance of these cells in the dialogue between external signals and the intestinal nervous or immunological regulations, this paracellular permeability would certainly be of particular importance in maintaining intestinal homeostasis in a cross‐talk between the lamina propria and the intestinal surface. It would be interesting to use this methodology to investigate paracellular permeability in the human intestine in its communities or distinctions.

## Author Contributions

Christine Coméra conceived and designed the research. Mathilde Miquel, Kadirey Verwaerde, Anissa Edir‐Kibri, Mikael Albin, Florence Blas‐Y‐Estrada, Audrey Samper, Elodie Rousseau‐Bacquie, and Christine Coméra performed the research and acquired the data. Mathilde Miquel, Kadirey Verwaerde, and Christine Coméra analyzed and interpreted the data. Hélène Eutamène, Vassilia Théodorou and Christine Coméra were involved in drafting and Christine Coméra in revising the manuscript.

## Conflicts of Interest

The authors declare no conflicts of interest.

## Supporting information


**Figure S1:** Identification of FM permeable cells in the jejunum of mice controls (A), or after atropine (B) or CCh (C) in vivo treatment. (D) Cell quantification/vilus of FM positive cells (Tot FM+), total GC (Tot GC) and FM positive GC (GC FM+) in ctrl (ctrl) or atropine or CCh treated mice (means ± SD for 45 villi/condition). Bars 50 μm.

## Data Availability

The data has been stored in the repository, openly available at https://doi.org/10.6084/m9.figshare.31007341.
