# Supplementary figures and images for: Mapping Intestinal Paracellular Perm Eability in Mice: Regional and Cellular Variability Under Physiological and Stimulated Conditions
Source: FASEB Bioadv. 2026 Mar 5;8(3):e70094. doi: 10.1096/fba.2025-00325 (PMC12963463; doi:10.1096/fba.2025-00325)

Figure S1

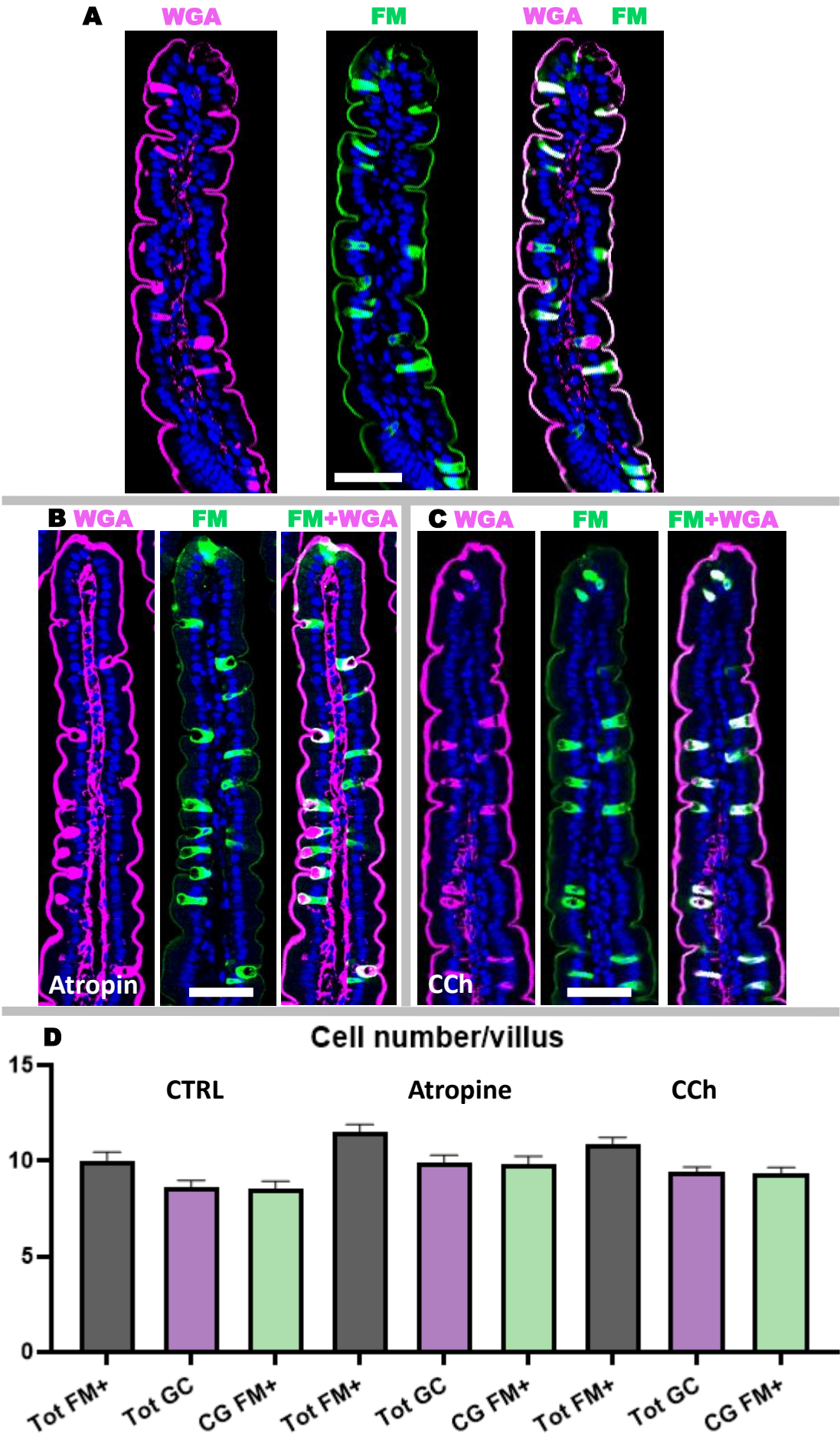

Supplement: Supplementary file 1 — Figure S1: Identification of FM permeable cells in the jejunum of mice controls (A), or after atropine (B) or CCh (C) in vivo treatment. (D) Cell quantification/vilus of FM positive cells (Tot FM+), total GC (Tot GC) and FM positive GC (GC FM+) in ctrl (ctrl) or atropine or CCh treated mice (means ± SD for 45 villi/condition). Bars 50 μm. [file FBA2-8-e70094-s001.pdf]
